# Serum Neopterin Concentration and Impaired Glucose Metabolism: Relationship With β-Cell Function and Insulin Resistance

**DOI:** 10.3389/fendo.2019.00043

**Published:** 2019-02-28

**Authors:** Jie-Eun Lee, Tae Jung Oh, Jae Hoon Moon, Kyong Soo Park, Hak Chul Jang, Sung Hee Choi

**Affiliations:** ^1^Department of Internal Medicine, Seoul National University Bundang Hospital, Seongnam, South Korea; ^2^Department of Internal Medicine, Seoul National University Hospital, Seoul, South Korea

**Keywords:** neopterin, kynurenine pathway, insulin resistance, β-cell function, impaired glucose metabolism

## Abstract

**Aim:** The purpose of this study was to measure the serum neopterin according to glucose metabolism and to evaluate neopterin as a predictor of type 2 diabetes (T2D) in a hospital-based cohort.

**Methods:** A 75-g oral glucose tolerance test (OGTT) was performed by people who visited the outpatient clinic in Seoul National University Bundang Hospital for suspected abnormal glucose tolerance or a strong family history of T2D. Neopterin was measured using an enzyme-link immunosorbent assay with baseline samples from the OGTT.

**Results:** Neopterin was measured in 184 participants. Indices related to glucose metabolism, such as the HOMA-IR, disposition index, etc. were calculated based on the results of the OGTT. The classifications for the 184 participants were: 24 (13%) had NGT, 89 (48.4%) prediabetes, and 60 (38.6%) T2D. Neopterin increased with deterioration of glucose metabolism (0.55 ± 0.25 vs. 0.58 ± 0.27 vs. 0.67 ± 0.27 ng/ml, *p* = 0.041; NGT, prediabetes, and T2D, respectively). Neopterin also correlated with fasting plasma glucose, 30-min and 120-min glucose of OGTT and HbA1c (*r* = 0.251, 0.259, 0.184, and 0.270, all *p* < 0.05). The HOMA-IR and disposition index correlated with neopterin (*r* = 0.291 and −0.170, respectively, both *p* < 0.05). When combined with C-peptide level, neopterin was as powerful as HOMA-IR in predicting future T2D.

**Conclusion:** Serum neopterin appears to be related to impaired insulin secretion and insulin resistance in the development of T2D. Further investigation of the relationship between neopterin and glucose metabolism would be helpful to understand the pathophysiology for the development of T2D.

## Introduction

Type 2 diabetes (T2D) is an important chronic disease because of the large number of people with T2D and its severe complications, which are responsible for increased morbidity and mortality. T2D is a complex metabolic disorder, and its pathogenesis is thought to have several mechanisms (1). One commonly held view is that risk factors, such as obesity, genetic factors, aging, and metabolic syndrome result in lipolysis and increased levels of circulating non-essential fatty acids. These factors can cause insulin resistance and lead to hyperglycemia, which has a further effect in reducing insulin secretion via “glucose toxicity” (2).

In 1997 and 1998, a new concept for the pathogenesis of T2D was proposed, which is that chronic inflammation and the innate immune system were closely involved (3, 4). Since then, many studies have shown that circulating markers of inflammation and acute-phase reactants are strong predictors of the development of T2D (5–9). In addition, there were reports of the role of adaptive immunity in obesity and related disease, such as insulin resistance in recent years (10–14). One implication of the results of these studies is that there may be a relationship between inflammation and diabetes; if so, inflammatory markers may be useful for refining T2D risk prediction and better targeting of individuals for lifestyle interventions.

Impaired glucose tolerance is one of the major risk factors for T2D, and the annualized conversion rate to T2D is 5–10% (15). Understanding the mechanisms through which prediabetes develops into T2D is important for the prevention and treatment of T2D. Inflammation is a prominent risk factor for the development of T2D in people with prediabetes (16). Neopterin, which is produced by activated macrophages/monocytes primarily upon stimulation by interferon-γ (IFN-γ), is a potential marker of immune activation.

At present, serum neopterin level is used primarily for diagnosing or predicting the prognosis of various diseases. The neopterin levels in body fluids are elevated by infection, autoimmune disease, malignancy, allograft rejection, cardiac and renal failure, coronary artery disease, and myocardial infarction (17). Neopterin is also known to be a byproduct of the kynurenine pathway, which is a metabolic pathway leading to the production of nicotinamide adenine dinucleotide (NAD) from the degradation of the essential amino acid tryptophan. The clinical and experimental data on the involvement of tryptophan metabolites in the pathogenesis of T2D were reviewed in 1985 (18). Pro-inflammatory factors, such as IFN-γ or lipopolysaccharide activate the upstream steps of the kynurenine pathway (i.e., conversion of tryptophan into kynurenine and kynurenine into 3-hydroxykynurenine) through activation of tryptophan—or indoleamine-2,3-dioxygenases and of kynurenine monooxygenase (19). These pro-inflammatory factors also divert downstream of the kynurenine pathway from the biosynthesis of NAD toward formation of diabetogenic downstream metabolites, such as kynurenine, xanthurenic acid, and kynurenic acid (20, 21). Concurrently with induction of indoleamine-2,3-dioxygenases, proinflammatory factors (e.g., IFN-γ, tumor necrosis factor-α, interleukin-1β) activate guanosine triphosphate cyclohydrolase, which catalyzes conversion of guanosine triphosphate to 7,8-dihydroneopterin (22) and results in the increased formation of a stable, water-soluble neopterin (23).

Many lines of evidence have shown that activation of the immune system and chronic inflammation are involved in the pathogenesis of T2D. Neopterin may be a possible biomarker for predicting T2D. The aim of our study was to measure the serum concentration of neopterin according to glucose metabolism status and to determine whether neopterin concentration is a predictor of future T2D in a hospital-based cohort.

## Materials and Methods

### Study Population and Diagnosis of T2D

A total of 184 subjects were randomly selected from a hospital-based cohort of patients who visited the outpatient clinic of the endocrinology department at Seoul National University Bundang Hospital (SNUBH) from 2005 to 2014 because of suspected abnormal glucose tolerance or a family history of T2D. They undertook a 75-g oral glucose tolerance test (OGTT), and blood glucose level was measured at 0, 30, and 120 min. The study participants were categorized according to the criteria for the diagnosis of T2D in the 2016 American Diabetes Association (ADA) guidelines (24). NGT was defined as a fasting plasma glucose (FPG) concentration < 100 mg/dl, 2-h post-prandial glucose (PG) < 140 mg/dl, and glycated hemoglobin (HbA1c) < 5.7%. Prediabetes was defined as FPG 100–125 mg/dl, 2-h PG 140–199 mg/dl, or HbA1c 5.7–6.4%. T2D was defined as FPG ≥ 126 mg/dl, 2-h PG ≥ 200 mg/dl, or HbA1c ≥ 6.5%. In the T2D group, newly diagnosed, drug-naïve patients were enrolled. Information about concomitant disease, medical history, and family history were collected through a retrospective review of medical records. People with pancreatic cancer, gestational diabetes, or type 1 diabetes, or those who used an insulin or oral hypoglycemic agent were excluded from the study. It was also confirmed whether there were inflammatory diseases, such as rheumatoid arthritis or infection known to affect the concentration of neopterin. The study was approved by the Institutional Review Board (IRB) of SNUBH (IRB No. L-2016-480-4) and complied with the Declaration of Helsinki (version 2008). Informed consent was waived by IRB because of the retrospective nature of the study.

### Laboratory Measurements

Venous blood was sampled after an 8-h fast. Samples were stored at −80°C immediately after blood withdrawal. Plasma glucose level was measured using a Hitachi 747 chemistry analyzer (Hitachi, Tokyo, Japan). HbA1c level was determined using a Bio-Rad variant II Turbo HPLC analyzer (Bio-Rad, Hercules, CA, USA) in the National Glycohemoglobin Standardization Program (NGSP) level II-certified laboratory at SNUBH. Plasma C-peptide and insulin concentrations were measured by radioimmunoassay (Linco, St. Charles, MO, USA). The fasting plasma concentration of total cholesterol, triglyceride, and high-density lipoprotein (HDL) and low-density lipoprotein (LDL) cholesterol were measured using a Hitachi 747 chemistry analyzer. Neopterin concentration was measured using commercially available ELISA kit (IBL International, Germany) in baseline samples from the OGTT. HbA1c, serum glucose, C-peptide, and insulin levels were measured in fresh plasma and neopterin concentration was assessed in frozen plasma.

### Indices of β-Cell Function, Insulin Sensitivity, and Insulin Resistance

The homeostasis model assessment of insulin resistance (HOMA-IR) index was calculated as fasting insulin (μU/ml) × FPG (mg/dl)/405 as described by Matthews et al. (25). HOMA-β was calculated using the following formula: 360 × fasting insulin concentration (μU/ml)/[FPG (mg/dl) – 63]. The 30-min insulinogenic index, an indicator of early phase insulin release, was calculated as [30-min insulin concentration (μU/ml)– fasting insulin concentration (μU/ml)]/[30-min glucose concentration (PP30) (mg/dl) – FPG (mg/dl)] (26). The disposition index, which reflects β-cell function, was calculated as [30-min insulin concentration (μU/ml) – fasting insulin concentration (μU/ml)]/[PP30 (mg/dl) – FPG (mg/dl)]/HOMA-IR (27, 28). The mean glucose and insulin concentrations were calculated from values at 0, 30, 120 min of the OGTT using the trapezoidal rule (29). The ratio of fasting glucose to insulin concentrations was measured to assess insulin sensitivity (30).

### Statistical Analysis

The data are expressed as mean with standard deviation (SD). All participants were categorized into three groups according to the 2016 ADA guidelines: NGT, prediabetes, and T2D. Significant differences in baseline characteristics were identified using the chi-squared test for categorical variables and Student's *t*-test or the Mann–Whitney *U* test for continuous variables. The baseline characteristics were analyzed according to the glucose metabolism status. The concentration of serum neopterin was square root transformed to achieve normal distribution and homogeneity of residuals. The relationships between neopterin concentration and the glucose-related parameters were analyzed using Pearson's correlation. Receiver operating characteristic curve (ROC) analysis (c-statistics) was used for predicting T2D, and differences in the area under the curve (AUC) between models were tested using the non-parametric method of DeLong et al. (31). In general, *p*-values < 0.05 were considered to indicate significant associations. Statistical data analyses were performed using IBM SPSS Statistics (version 22.0; IBM Corp., Armonk, NY, USA).

## Results

### Patient Characteristics

A total of 184 participants aged 21–88 years were included. Twenty-four (13%) were classified as having NGT, 89 (48.4%) as having prediabetes, and 60 (38.6%) as having T2D. Their baseline characteristics are shown in Table 1. Their mean age was 52.1 ± 12.3 years, and their mean BMI was 25.0 ± 3.8 kg/m^2^. For all participants, the median neopterin concentration was 0.366 ng/ml (interquartile rage, 0.156–0.627). Neopterin concentration did not differ significantly according to age, sex or smoking history (data not shown), but was significantly related to BMI (*r* = 0.201, *p* < 0.05). Neopterin concentration was also significantly higher in patients with T2D than in those with NGT or prediabetes but did not differ significantly between the NGT and prediabetes groups (Table 1). White blood cell count and C-reactive protein concentration, a clinical marker of inflammation, were higher in patients with T2D than in those with NGT.

**Table 1:** Baseline characteristics of the study participants.

	**NGT**	**Prediabetes**	**T2D**	***p***
	**Mean ± SD**	**Mean ± SD**	**Mean ± SD**	
Age, years	47.8 ± 13.6	56.8 ± 9.8	47.5 ± 12.6	< 0.001
BMI, kg/m^2^	23.4 ± 2.7	24.5 ± 3.0	26.2 ± 4.6	0.002
SBP, mmHg	123.6 ± 15.4	125.1 ± 14.5	122.8 ± 14.4	0.646
DBP, mmHg	73.4 ± 7.2	76.3 ± 9.9	78.3 ± 9.5	0.191
Cholesterol, mg/dl	196.6 ± 24.9	195.2 ± 36.2	213.0 ± 45.5	0.016
Triglyceride, mg/dl	134.4 ± 120.6	153.4 ± 150.2	202.2 ± 197.8	0.126
HDL, mg/dl	58.1 ± 16.4	53.2 ± 13.7	48.1 ± 11.2	0.005
LDL, md/dl	102.5 ± 25.6	107.1 ± 31.3	125.3 ± 31.6	0.001
HbA1c, %	5.3 ± 0.2	6.0 ± 0.6	10.8 ± 2.0	< 0.001
FPG, mg/dl	91.3 ± 6.4	111.1 ± 19.0	225.8 ± 88.3	< 0.001
PP30, mg/dl	146.8 ± 23.8	195.0 ± 32.1	330.7 ± 101.9	< 0.001
PP2, mg/dl	103.0 ± 17.7	151.4 ± 38.1	397.6 ± 117.1	< 0.001
Basal C-peptide, ng/ml^§^	1.6 ± 0.7	1.9 ± 0.8	2.1 ± 1.1	0.037
Basal insulin, μIU/ml	10.6 ± 3.3	11.2 ± 4.4	12.3 ± 6.5	0.253
Insulin30, μIU/ml	56.9 ± 25.2	45.4 ± 26.3	17.4 ± 9.5	< 0.001
Insulin120, μIU/ml	38.0 ± 25.5	54.7 ± 39.2	23.7 ± 17.6	< 0.001
Insulinogenic index	0.97 ± 0.72	0.39 ± 0.47	0.06 ± 0.09	< 0.001
HOMA-IR	2.41 ± 0.83	3.05 ± 1.27	6.90 ± 4.43	< 0.001
Disposition index	0.45 ± 0.34	0.14 ± 0.20	0.01 ± 0.02	< 0.001
HOMA-β	141.23 ± 42.92	93.27 ± 40.79	32.74 ± 29.03	< 0.001
Glucose:insulin ratio	9.39 ± 3.04	11.68 ± 6.45	22.62 ± 14.49	< 0.001
WBC, count/mm^3^	5.5 ± 1.8	6.0 ± 1.6	7.1 ± 2.0	< 0.001
hs-CRP	0.370 ± 1.560	0.130 ± 0.220	0.730 ± 2.020	0.042
Neopterin, ng/ml^§^	0.545 ± 0.253^a^	0.577 ± 0.274^b^	0.672 ± 0.268	0.041

§ *Square root transformed*.

a *vs. DM, p = 0.048*.

b *vs. DM, p = 0.028*.

*NGT, normal glucose tolerance; T2D, type 2 diabetes; BMI, body mass index; SBP, systolic blood pressure; DBP, diastolic blood pressure; HDL, high density lipoprotein; LDL, low density lipoprotein; HbA1c, glycated hemoglobin; FPG, fasting plasma glucose, PP30, 30-min glucose concentration; PP2, 2-h glucose concentration; HOMA-IR, homeostatic model assessment-insulin resistance; HOMA-β, homeostatic model assessment-β cell; WBC, white blood cell; hs-CRP, high-sensitive C-reactive protein*.

### Associations Between Serum Neopterin Concentration and Glucose Metabolic Status

Serum neopterin correlated significantly with FPG (*r* = 0.251, *p* < 0.001), with PP30 and PP2 of the 75-g OGTT (*r* = 0.259 and 0.184, respectively, both *p* < 0.05), and with HbA1c level (*r* = 0.270, *p* < 0.001) (Table 2). These correlations with serum glucose levels remained significant after adjusting for age and BMI. However, serum neopterin showed a significant correlation only with baseline insulin, not with 30-min or 120-min insulin concentration of OGTT, which was not maintained after adjustment for age and BMI. For the glucose metabolism-related parameters, serum neopterin correlated positively with HOMA-IR (*r* = 0.291, *p* < 0.001) and inversely with the disposition index after adjusting for age and BMI (*r* = −0.162, *p* < 0.05). However, HOMA-β and insulinogenic index were not significantly correlated with serum neopterin (data not shown).

**Table 2:** Partial correlations between serum neopterin concentration and glucose metabolism-related parameters.

	**Unadjusted**		**Adjusted for age and BMI**	
	***r***	***p***	***r***	***p***
HbA1c, %	0.270	<0.001	0.296	0.002
FPG, mg/dl	0.251	0.001	0.268	0.004
PP30, mg/dl	0.259	<0.001	0.258	0.006
PP2, mg/dl	0.184	0.014	0.214	0.023
Basal C-peptide, ng/ml	0.251	0.001	0.242	0.010
Basal insulin, μIU/ml	0.184	0.013	0.109	0.251
HOMA-IR	0.291	<0.001	0.258	0.006
Disposition index	−0.170	0.021	−0.162	0.032

*The data were analyzed using Pearson correlational analysis*.

*Neopterin concentration was square root transformed*.

*BMI, body mass index; FPG, fasting plasma glucose, PP30, 30-min glucose concentration; PP2, 2-h glucose concentration; HOMA-IR, homeostatic model assessment-insulin resistance*.

### Relationship Between T2D Progression and Serum Neopterin Concentration

Of the 184 participants, 108 were followed up for more than 6 months; the mean follow-up period was 56.94 ± 32.83 months. During the follow-up, 39 patients progressed to T2D and 69 patients did not progress to T2D. Serum neopterin concentration did not differ significantly between those who progressed and those who did not progress to T2D (data not shown). The AUC value was 0.524 when the c-statistics were calculated using only neopterin concentration to predict the occurrence of T2D. However, model 3 (shown in Table 3), in which neopterin and C-peptide concentrations were considered together, gave an AUC value similar to that of HOMA-IR for predicting future T2D (AUC 0.649 and 0.624, respectively, *p* = 0.640).

**Table 3:** Comparison of AUCs for predicting the occurrence of type 2 diabetes.

	**AUC**	**SE**	**95% CI**	***p*-value (vs. model 1)**	***p*-value (vs. model 2)**	***p*-value (vs. model 3)**	***p*-value (vs. model 4)**
Model 1	0.524	0.0601	0.423–0.623				
Model 2	0.609	0.0584	0.508–0.704	0.391			
Model 3	0.649	0.0552	0.549–0.741	0.105	0.244		
Model 4	0.624	0.0572	0.523–0.717	0.284	0.766	0.640	
Model 5	0.685	0.0528	0.586–0.773	0.029	0.327	0.620	0.339

*Model 1, neopterin concentration; Model 2, basal C-peptide concentration; Model 3, basal C-peptide + neopterin concentrations; Model 4, HOMA-IR; Model 5, disposition index*.

## Discussion

In the present study, we found that serum neopterin concentration was significantly associated with glucose metabolism-associated parameters, such as HOMA-IR, disposition index as well as serum glucose level and basal C-peptide. It was higher in patients with T2D than in those with NGT or prediabetes. In addition, considering both serum neopterin and C-peptide concentrations had a similar power to that of HOMA-IR in predicting the development of T2D.

A common feature of insulin resistance-related disorder, such as T2D is low-grade inflammation, which may be indirectly measured by the levels of neopterin or kynurenine metabolites (32–35). Inflammatory processes are involved in the progression of insulin resistance and β-cell dysfunction in individuals with prediabetes and contribute to the development of diabetes (36). It has been previously reported that serum neopterin level is associated with insulin resistance in a couple of population-based cohort studies (37, 38). The current report confirmed its association with insulin resistance (HOMA-IR) as well. Moreover, we found that serum neopterin level have a significant correlation with disposition index, a measure of acute insulin secretion of β-cell function. Neopterin or kynurenine metabolites were supposed to be related to β-cell function through the activation of apoptotic caspase, etc. (39–42). Considering the mechanisms responsible for the increased levels of neopterin or kynurenine metabolites, they are expected to increase in relation to T2D.

Increased kynurenine metabolites have been reported in the urine of rat and monkey models of diabetes (43). An elevation in the concentration of kynurenine pathway metabolites has been shown in human serum and urine samples of diabetic patients in comparison with that of non-diabetic controls (43, 44). On the contrary, neopterin was not higher in the diabetes group compared to the control group in a couple of population-based cohorts (45, 46). These different outcomes in patients with diabetes may be due to uncontrolled factors that known to be potentially affect serum neopterin levels, such as age, BMI, hypoglycemic agents or diabetic complications. In this study, we evaluated the newly diagnosed patients with T2D who did not take any hypoglycemic agents and it can be considered the most reliable results to date by minimizing these effects.

Given the association of serum neopterin concentration, insulin resistance, and β-cell function, such as insulin secretion, we explored the serum neopterin concentration as a predictor of the progression to T2D. Among the 108 participants who were followed up for 6 months or more, there was no significant difference in serum neopterin concentration between progressors to future T2D and non-progressors. In the C-statistics analysis for the occurrence of T2D with neopterin alone, the AUC was insignificantly as low as 0.524 [95% confidence interval (CI), 0.423–0.623].

However, when both serum neopterin and C-peptide concentrations were considered together, the AUC value was 0.649 (95% CI, 0.549–0.741), which was similar to that of the HOMA-IR (0.624, 95% CI, 0.523–0.717) for predicting T2D. We can speculate that these results reflect the characteristics of Asian patients in the pathogenesis of diabetes. As is well-known, Asians have a higher risk of developing diabetes due to impaired insulin secretion which was induced by insufficient pancreatic β-cell mass or functional defects (31). Therefore, further studies are needed to clarify the mechanism of neopterin in the pathogenesis of the progression of diabetes in conjunction with the impaired beta cell function.

This study has some limitations. First, a prospective follow up study was conducted on limited number of study subjects. Only about half of the subjects were followed up for more than 6 months, and 35% of these subjects were progressed to T2D. Therefore, statistically significant result may not be obtained, especially in the comparative analysis of neopterin depending on the progression to T2D. 14 Second, the prediabetes group included people with impaired fasting glucose (IFG), impaired glucose tolerance (IGT), or both. IFG and IGT are different with respect to the degree of insulin resistance and insulin secretion (47), and it is possible that serum neopterin concentration may differ between IFG alone, IGT alone, and IFG+IGT together in large study subjects. Despite these limitations, our study had several strengths, the major one being that it is the first study to evaluate serum neopterin concentration according to glucose metabolism status with 75g OGTT (NGT, prediabetes, and T2D) in 184 participants. In addition, all patients diagnosed with T2D were drug-naïve, and blood samples were collected before the administration of medication. In 1989, Marchetti et al. reported a relationship between metformin level and plasma amino acid concentration, specifically that metformin might elevate plasma tryptophan level (48), which might affect the concentration of serum neopterin level even to a small extent. Therefore, it is important that we were able to exclude the effect of oral hypoglycemic agents by including only drug-naïve patients.

In conclusion, we found that serum neopterin concentration was closely related to glucose intolerance. As the predictor for future T2D, serum neopterin alone was not an independent factor, but it is significant when it considered with c-peptide level together. Further investigation of the relationship between serum neopterin concentration and glucose metabolism in a large study population would be helpful to understand the pathophysiology for the development of T2D.

## Author Contributions

J-EL and SC designed the study. J-EL, TO, JM, KP, HJ, and SC contributed data interpretation. J-EL and SC collected and analyzed the data, and drafted the manuscript. All authors agreed on the final content of the manuscript.

### Conflict of Interest Statement

The authors declare that the research was conducted in the absence of any commercial or financial relationships that could be construed as a potential conflict of interest.
